# Electromagnetic Shielding and Flame Retardancy of Composite Films Constructed with Cellulose and Graphene Nanoplates

**DOI:** 10.3390/ma15031088

**Published:** 2022-01-30

**Authors:** Zuwei Fan, Yuanyuan Yu, Xiaojie Cheng, Rangtong Liu

**Affiliations:** College of Fashion Technology, Zhongyuan University of Technology, Zhengzhou 451191, China; yyyamanda@163.com (Y.Y.); cxj_cheng@126.com (X.C.); ranton@126.com (R.L.)

**Keywords:** composite film, cellulose, graphene nanoplates, electromagnetic shielding, flame retardancy, lamination

## Abstract

Aimed at improving the electromagnetic (EM) shielding and flame retardancy of cellulose materials, graphene (GE) nanoplates were introduced into cellulose matrix films by blending in 1-allyl-3-methylimidazolium chloride. The structure and performance of the obtained composite films were investigated using scanning electron microscopy, X-ray diffraction, thermogravimetric (TG) analysis, EM shielding effectiveness (SE), and combustion tests. GE introduction formed and stacked laminated structures in the films after drying due to controlled shrinkage of the cellulose matrix. The lamination of GE nanoplates into the films was beneficial for providing EM shielding due to multiple internal reflection of EM radiation; furthermore, they also increased flame resistance based on the “labyrinth effect.” The SE of these composite films increased gradually with increased GE content and reached 22.3 dB under an incident frequency of 1500 MHz. TG analysis indicated that these composite films possessed improved thermal stability due to GE addition. Reduced flammability was confirmed by their extended times to ignition or inability to be ignited, reduced heat release rates observed in cone calorimetry tests, and increased limiting oxygen index values. These films with improved EM shielding and flame retardancy could be considered potential candidates for multipurpose materials in various applications, such as electronics and radar evasion.

## 1. Introduction

Biobased materials have become promising alternatives to synthetic polymers due to their nontoxic nature and renewability [1]. Currently, many biobased materials, such as cellulose, chitin, and keratin, have been developed and widely used in textiles, packaging, biomedicines, smart devices [2,3,4], and so on. Cellulose, derived from plants, is the most abundant natural polymer on earth. In contrast with synthetic polymers, natural cellulose cannot be heated, melted, or dissolved in common solvents; therefore, it has been used in primitive form for most situations until recent decades. Since the development of effective solvents, hydrogen (H-) bonds between cellulose chains can be destroyed and rearranged to form membranes, films, fibers, hydrogels, aerogels, and other materials [5,6,7,8,9].

Due to differences with traditional metals and their alloys, polymers are gradually attracting increasing attention in electromagnetic (EM) shielding applications because of their unique characteristics, such as being lightweight, flexible, and corrosion resistant [10]. At present, polymer-based EM shielding composite materials mainly contain surface-conductive types and filled types. Surface-conductive materials are obtained by attaching a thin conductive layer to the surface of the polymer base, thereby achieving EM shielding properties [11,12]. Surface-conductive materials show good conductivity and can reflect EM waves. Filled polymer-based materials are usually obtained by blending conductive components with a polymer matrix. These materials not only exhibit characteristics of fillers but they also inherit advantages of polymers, such as low density, easy processing, and corrosion resistance. Moreover, a wide range of conductive fillers and polymer types can be selected according to a scenario’s requirements. Currently, the choices of conductive fillers include metal fillers [13,14], carbon-based fillers [15,16], intrinsically conducting polymer fillers [17,18], and composite conductive fillers [19,20].

As a natural organic polymer, cellulose has insulation qualities but is also flammable, which limits its application in some fields. In order to overcome these problems, various forms of regenerated cellulose have been coated with polypyrrole, via in situ oxidative polymerization or chemical vapor deposition, to fabricate conductive composites [21,22,23]. In some cases, such materials can provide EM reflection due to their conductive coating layers; in addition, their surface coatings may serve, to some extent, as fire retardants. However, these fabrication methods often require special facilities and complicated processes; moreover, the interfacial bonding between coating layers and cellulose templates is too weak for some special application environments.

The regeneration of cellulose has been achieved through dissolution in special solvents, such as *N*-methylmorpholine oxide [24], ionic liquids [25], and some mixed solvents [26]. Therefore, some functional materials have been introduced into regenerated cellulose materials by solution mixing. A graphite, powder-embedded, cellulose composite film was prepared by Chen et al. [27], in which graphite powder was dispersed in an ionic liquid before cellulose dissolution. Graphite incorporation improved thermal stability as well as increased electrical conductivity, resulting in excellent EM interference (EMI) and shielding effectiveness (SE). However, film with 200% graphite added burned into fragments during combustion; the embedded graphite remained, while the cellulose was completely thermally degraded. Graphene (GE) derives from the exfoliation of graphite, and it is a flat sheet of carbon that is only one-atom thick. It has been intensively investigated as a new type of carbon-based nanomaterial due to its advantages of ultrahigh surface area, excellent mechanical flexibility, thermal stability, and electrical conductivity [28,29,30]. These outstanding features make graphene an excellent option for improving conductivity, EM shielding, and flame retardancy of cellulose materials. Some cellulose/GE films with excellent EMI shielding performance have been fabricated by vacuum filtration of cellulose nanofiber/GE suspensions [31,32] or by employing GE to decorate porous cellulose aerogels [26]. Based on the above-mentioned solution regeneration method, lotus fiber/GE composite films have been prepared by Cheng et al. [33]. The resulting composite films showed high electrical conductivity, excellent EMI SE, and an ability to generate heat. However, the effects of GE introduction on film flammability have not been investigated. Moreover, pure cellulose has not been used and studied, as lotus fiber contains cellulose and related derivatives.

In this study, GE nanoplates with well-ordered arrangements were introduced into cellulose films to fabricate a multifunctional material with excellent EM shielding and flame retardancy. GE nanoplates were added before cellulose dissolution in an ionic liquid, 1-allyl-3-methylimidazolium chloride ([Amim]Cl), that has been used for dissolving various natural cellulose fibers [33]. The resulting cellulose/GE composite films were obtained by regeneration using a coagulation bath and controlled drying. Structural characteristics of the obtained films were investigated by scanning electron microscopy (SEM), energy-dispersive spectrometry (EDS), and X-ray diffraction (XRD). Mechanical properties, conductivity, EMI SE, thermal stability, and flammability of these films were also investigated in detail. This study provided information regarding a natural cellulose matrix film with both excellent EM and thermal properties.

## 2. Experimental Section

### 2.1. Materials

Cellulose powders (α-cellulose; particle size, 90 μm) were purchased from Shanghai Macklin Biochemical Co., Ltd. (Shanghai, China). GE nanoplates (KNG-G2; 1–3 layers; flake size, 7–12 μm) were obtained from Xiamen Knano Graphene Technology Corp., Ltd. (Xiamen, China). [Amim]Cl (purity ≥99%) was provided by Shanghai Aichun Biological Technology Co., Ltd. (Shanghai, China).

### 2.2. Fabrication of Composite Films

**Figure 1:**
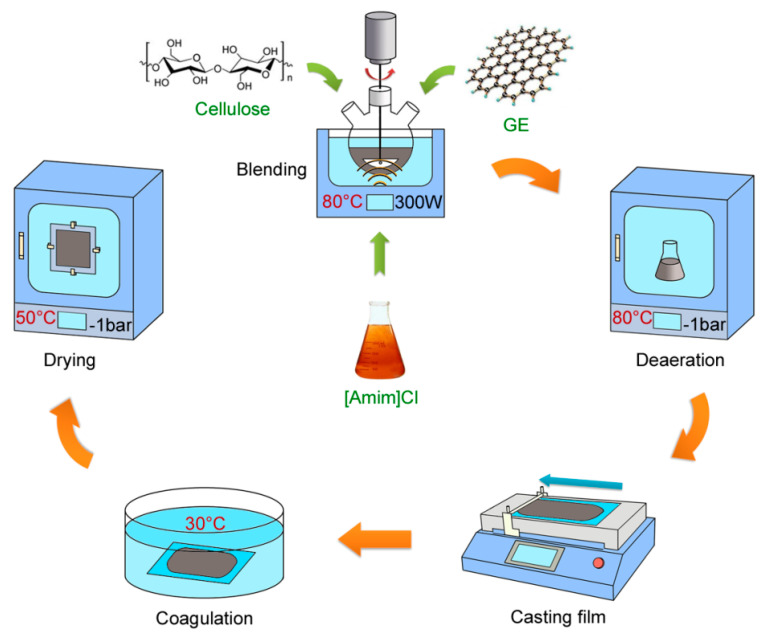
Schematic diagram of composite film fabrication process.

The fabrication procedure of the composite films is shown in Figure 1. Initially, GE dispersions with varying GE content (0–6 wt% relative to final mixed solution) were prepared by adding GE into [Amim]Cl, followed by magnetic stirring along with ultrasonic vibrations at 80 °C for 6 h. Subsequently, cellulose powders at 3 wt% were added into the dispersions, and the mixtures were finally obtained after continuous magnetic stirring at 80 °C for 6 h. Each obtained solution was degassed under vacuum at 80 °C for 12 h; then, they were cast on a glass plate with a thickness of 2 mm via a coating machine. Immediately, the solution layer along with a glass plate was immersed into a 30 °C water bath to coagulate and regenerate. Then, the regenerated films were washed several times with pure water. Next, they were dried under vacuum at 50 °C while sandwiched between plastic frames to prevent shrinkage. The obtained films had GE contents of 0, 1.5, 3, 4.5, and 6 wt%, and they were named F0, F1, F2, F3, and F4, respectively.

### 2.3. Characterization

Micromorphology of the films and their elemental distributions were analyzed using SEM (Zeiss Supra 55, Carl Zeiss AG, Oberkochen, Germany) and associated EDS, respectively. All samples were fractured in liquid nitrogen and gold-sprayed prior to SEM observation. XRD patterns of the films were measured by using an XRD instrument (Smart Lab 9, Rigaku, Tokyo, Japan), with 45 kV X-rays, 200 mA, recorded from 10 to 50° (2*θ*), and 1°/min scanning speed.Thermogravimetric (TG) analysis was performed using a TG analyzer (TGA 8000, PerkinElmer Inc., Waltham, MA, USA) at a heating rate of 10 °C/min from 30 to 800 °C under a nitrogen atmosphere.

Film thickness was tested using a portable thickness gauge, and planar density was measured by weighing fixed-area film samples. Tensile strength and elongation at break were tested using an electromechanical universal testing machine (CMT4204, MTS System (China) Co., Ltd., Shenzhen, China). The films were cut into 1-centimeter-wide strips and used for tensile testing, in which the gripping range and tensile rate were 10 cm and 10 cm/min, respectively.

Electrical conductivity was assessed by conducting electrical resistance measurements. Using 1-centimeter-wide strips that are 10-centimeters long, the resistances of the samples were measured by using a modern digital multimeter (UT61E, Uni-Trend Technology (China) Co., Ltd., Dongguan, China). Their electrical conductivities were calculated according to Equation (1):

(1) $$\sigma =\frac{l}{R\cdot d\cdot \delta }$$

where *σ* and *R* represent the electrical conductivity (S/m) and resistance (Ω) of each strip, respectively; and *l*, *d*, and *δ* represent strip length, width, and thickness (m), respectively.

EMI SE of the films was measured using the flange coaxial method based on ASTM D4935-2010 standard. A fabric EM SE tester (DR-913G, Wenzhou Darong Textile Instrument Co., Ltd., Wenzhou, China) was employed, consisting of a vector network analyzer and a vertically lifting sample holder connected with a coaxial cable line, for testing SE over a frequency range of 50–3000 MHz.

Film flame resistance tests were carried out by igniting the lower ends of samplestrips with a 45-degree-tilt using a flame, followed by moving the flame away, and recording the film burning process. Combustion behaviors of the films under high-temperature conditions were also tested using a cone calorimeter (CCT, Motis Fire Technology (China) Co., Ltd, Kunshan, China) according to ISO 5660-1:2003 standard, which required sample sizes of 10 cm × 10 cm. The limiting oxygen index (LOI) was tested by using an apparatus (CO1, Motis Fire Technology Co., Ltd., Kunshan, China), in which each sample was 12 cm × 5.5 cm in size.

## 3. Results and Discussion

### 3.1. Structure and General Properties

**Figure 2:**
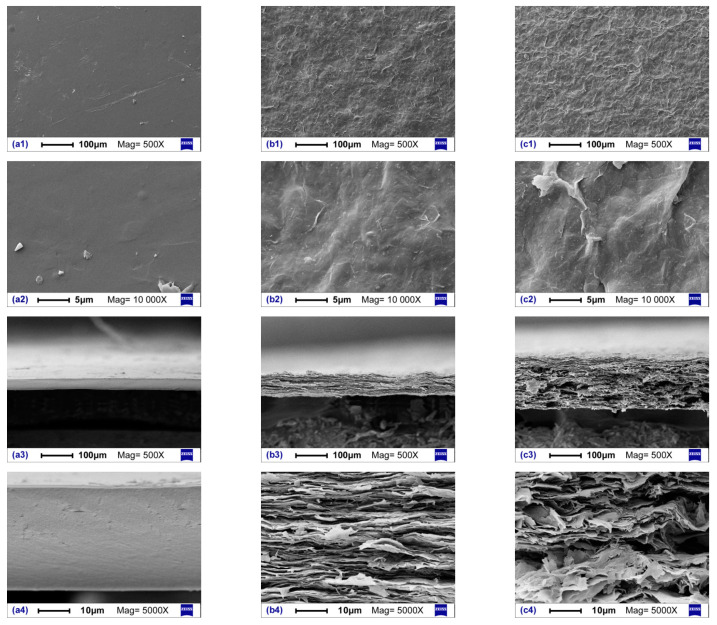
SEM images of cellulose and composite films: (**a**) F0, (**b**) F2, and (**c**) F4; (**1**,**2**) surface; and (**3**,**4**) cross-section.

After drying, pure cellulose film was smooth and transparent, while composite films with GE nanoplates appeared matte and black. The surfaces and cross-sectional micromorphologies of the cellulose and composite films were observed by SEM (Figure 2), and the results showed that F0 film possessed a smooth surface, but composite films F2 and F4 exhibited rough surfaces with wrinkles due to the embedded GE nanoplates. From cross-sectional views, dense structures were observed inside the F0 film; with GE introduction, film thicknesses increased and dense structures were replaced by stacked laminated structures. All wet films possessed similar large thicknesses (Table 1), but drying resulted in film shrinkage due to strong intermolecular and intramolecular H-bond actions of cellulose after water loss. Shrinkage mainly occurred in the thickness dimension because the film area was fixed during drying. GE addition raised the solid content in cast solutions such that the thicknesses and planar densities of the resulting dry films increased. Thickness shrinkage resulted in the formation of GE-laminated structures. The presence of GE restricted film shrinkage to some extent, which was observed from thickness differences between wet and dry films. Therefore, F4 film, having shrunk by a lesser degree, exhibited looser laminated structures than F2.

The effects of GE content on film mechanical properties were observed (Table 1). With the addition of GE nanoplates, film tensile strength clearly decreased and elongation slightly decreased. After drying, pure cellulose film possessed high strength and rigidity via H-bonds, while embedded GE agglomerations weakened H-bonding and reduced film strength. However, as a two-dimensional filler, GE nanoplates had some structural integrity, which ensured moderate strength (>10 MPa) of these composite films.

**Figure 3:**
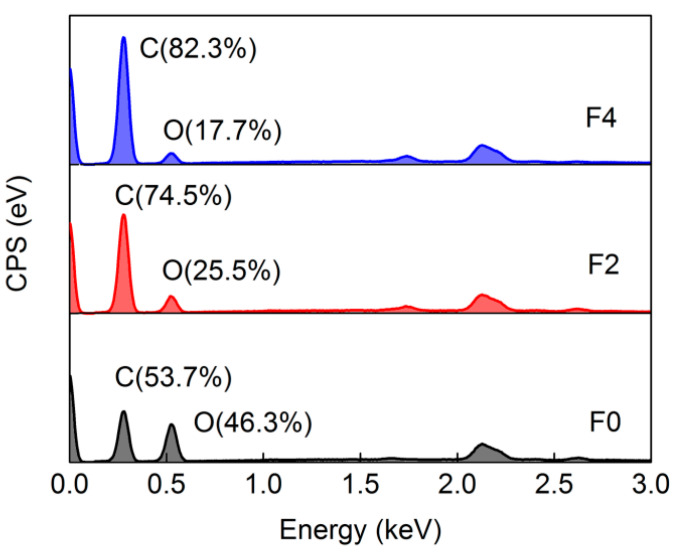
EDS patterns of cellulose and composite film surfaces.

**Figure 4:**
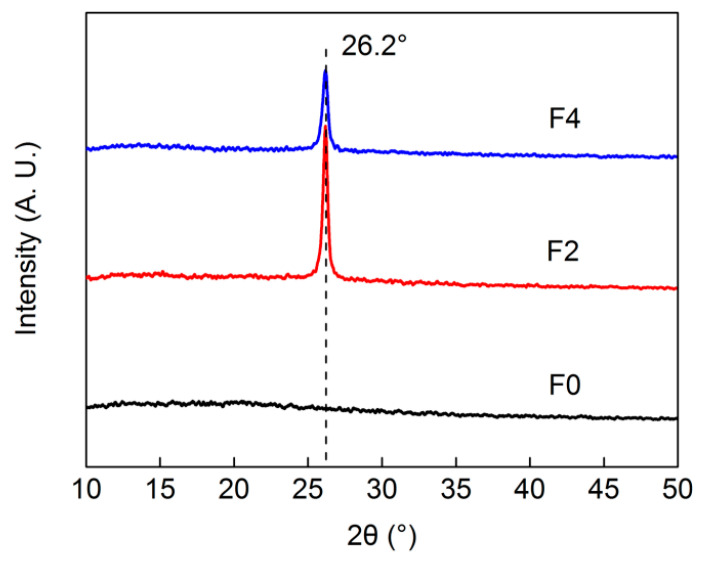
XRD patterns of cellulose and composite films.

The presence of GE in these composite films was further investigated using EDS and XRD examinations. According to elemental distributions of the film surfaces obtained from EDS analyses (Figure 3), pure cellulose film surfaces consisted of carbon (C) and oxygen (O); with GE introduction, the proportion of O decreased as a result of decreased cellulose and increased GE containing only C on film surfaces. From Figure 4, XRD patterns of these films, when 2*θ* ranged from 10° to 50°, showed that there were no clear characteristic peaks observed from the F0 film, which indicated that cellulose material comprised amorphous regions after dissolution and regeneration in [Amim]Cl [34]. Sharp peaks at 26.2° appeared in XRD patterns of all composite films, corresponding to the graphtic (002) face of embedded GE nanoplates [35] with less layering and regular lamination. In addition, the GE-derived peak in the XRD pattern of F4 film was observed to be weaker than that of F2, despite higher GE contents in F4. As mentioned above, the lower shrinkage degree in F4 resulted in looser and less-ordered laminated structures such that reflections from ordered agglomerations of the GE nanoplates were reduced.

### 3.2. Electromagnetic Shielding Performance

**Figure 5:**
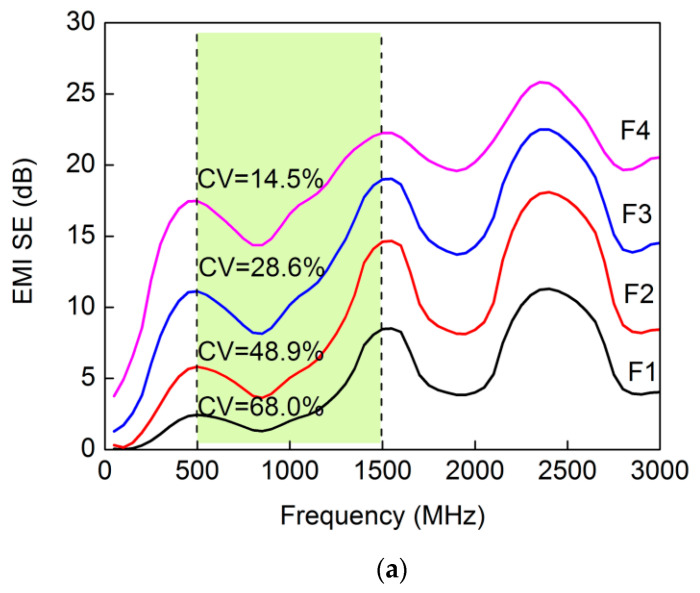

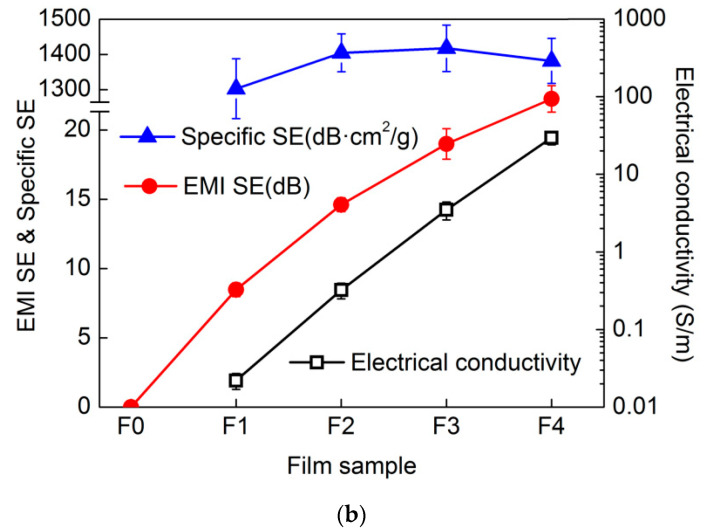
EM properties of composite films: (**a**) variation of EMI SE with incident frequency from 50 to 3000 MHz; (**b**) SE at 1500 MHz and electrical conductivity.

These composite films were endowed with EM shielding performance via the GE nanoplates, exhibiting excellent electrical conductivity; EMI shielding effects observed in the films were also affected by GE content. EMI SE curves of the various films, over an incident frequency range of 50–3000 MHz, showed that all composite films presented similar SE variation trends over this frequency range as well as observed regular fluctuations, with three peaks located at 500, 1500, and 2400 MHz (Figure 5a). Pure cellulose films, without GE, were transparent to EM waves, thus possessing an SE value of 0 dB. With GE nanoplates, composite film SE showed improvement over the entire frequency range. Typically, SE at 1500 MHz increased with increased GE content, exhibiting a regularly positive relationship (Figure 5b) and increasing to 22.3 dB for F4 film, which is considered an adequate and moderate level of EM shielding for many applications, as compared to some previously reported cellulose-based EM shielding films shown in Table 2. In order to investigate SE fluctuations with frequency, a frequency range from 500 to 1500 MHz was selected to calculate the coefficient of variation (CV), because EM shielding was weak at low frequencies, and SE measurements at frequencies above 1500 MHz would have been an extended test of the flange coaxial method. Increased GE content not only improved SE but also reduced CV from 68.0% (F1) to 14.5% (F4), which indicated that composite films with higher GE content had an SE with smaller fluctuations according to frequency and exhibited good EM shielding over a wider frequency range.

The EMI SE of a composite material is generally considered to be related to its electrical conductivity [36]. With increased GE content, the GE/cellulose ratio increased, which improved the conductivity of composite films after drying, since GE nanoplates touched one another more often to form a conductive network with better connectivity. Comparing the curves displayed in Figure 5b, composite film electrical conductivity was found to be closely related to EMI SE, yet it exhibited little impact on specific SEs (EMI SE divided by planar density of the composite film). Therefore, the shielding provided by composite films from EM radiation was not entirely determined by their electrical conductivity. EMI shielding performance of these films originated from a combination of the following two effects: on the one hand, EM energy was absorbed by GE nanoplates, resulting in energy dissipation of EM microwaves; on the other hand, reflections of EM radiation occurred on the surfaces of GE nanoplates, and multiple internal reflections occurred between the laminated GE nanoplates due to the inhomogeneity of scattering effects within the films. The laminated structure of GE nanoplates was more ordered in the F2 film than in the F4 film (Figure 2) such that its multiple internal reflection effects were better than those of F4, and its specific SE was slightly higher than F4 (Figure 5b). Moreover, cellulose was separated and protected between the laminated GE nanoplates, which was also beneficial for reducing composite film flammability (discussed below).

### 3.3. Thermal and Antiflaming Performance

**Figure 6:**
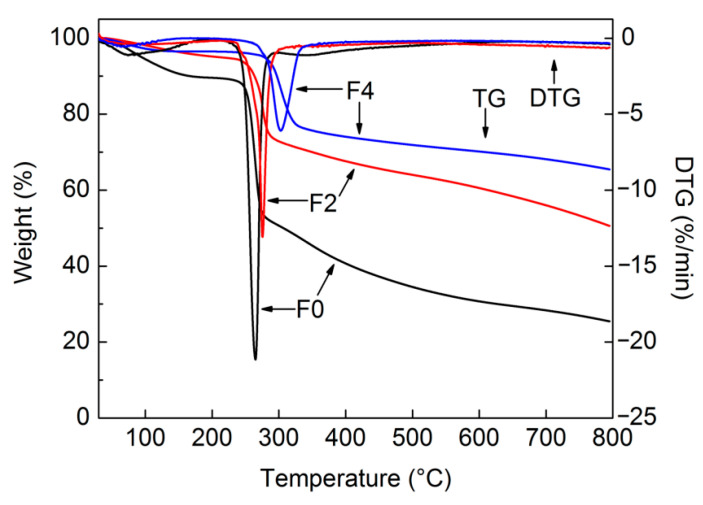
TG analysis of cellulose and composite films.

From TG analyses of the films, F0 exhibited relatively obvious initial weight loss from the evaporation of water into ambient air due to the hydrophilicity of pure cellulose; the most significant weight loss (36.4%) occurred at around 272 °C (Figure 6). However, maximum weight loss temperatures increased to 282 and 305 °C for F2 and F4, respectively, and their corresponding weight losses reduced to 20.3 and 19.1%, respectively. This was due to the addition of GE and the excellent thermal stability it provided to the cellulose matrix. Additionally, the residual film weights clearly increased when heated to 800 °C with GE, which was attributed to the increased percentage of indecomposable GE. GE-formed lamellar structures delayed the release of cellulose pyrolysis products such that thermal degradation temperatures of the composites slightly increased. TG analytical results indicated that these composite films exhibited improved thermal stability relative to pure cellulose film.

**Figure 7:**
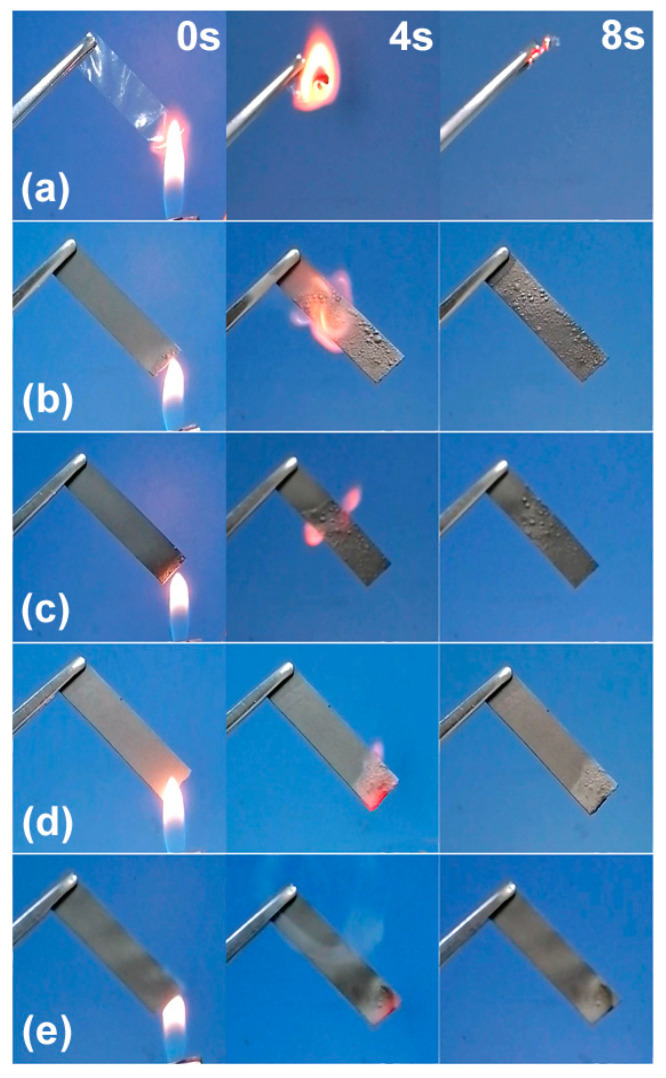
Combustion process of cellulose and composite films through ignition: (**a**) F0, (**b**) F1, (**c**) F2, (**d**) F3, and (**e**) F4.

Film flammability was initially evaluated by directly igniting sample strips by using a flame (Figure 7). F0 film was observed to ignite easily and burn up rapidly due to the flammability of natural cellulose. With GE introduction, the intensities and rates of combustion in the films were clearly reduced. In particular, the F3 film self-extinguished when taken away from the igniting flame, and the F4 film could not be ignited, thus exhibiting outstanding flame resistance. Moreover, in contrast with changes observed in pure cellulose film, composite films maintained their original shapes after combustion, since only some of the cellulose components within them burned.

**Figure 8:**
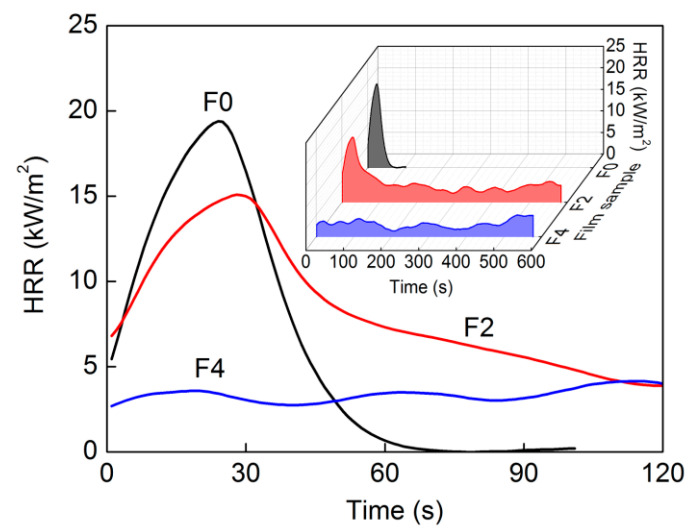
HRR curves of cellulose and composite films by cone calorimeter analysis.

**Figure 9:**
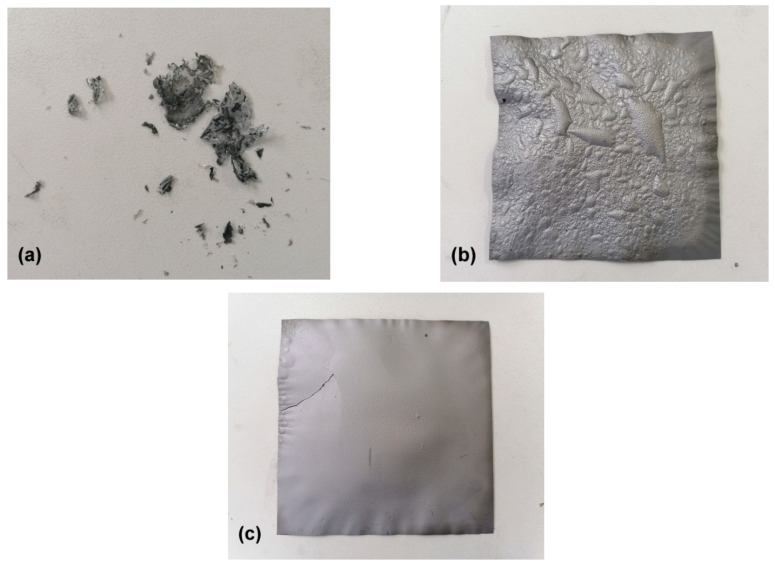
Photographs of samples (**a**) F0, (**b**) F2, and (**c**) F4 after cone calorimetry tests.

Cone calorimetry testing is an effective method for evaluating material flammability and serves as an important reference for flame-retardant mechanisms [37]. Heat release rate (HRR) curves from these films were collected to determine time to ignition (TTI), time to flameout (TTO), peak HRR (pkHRR), total heat release (THR), peak smoke production rate (pkSPR), and total smoke production (TSP) (Figure 8 and Table 3). Films exposed to heat radiation absorbed enough heat to produce combustible gases, which made them burn suddenly and release abundant heat, reaching pkHRR [38]. Comparing F0 and F2, GE introduction not only extended TTI from 5 to 10 s but also reduced pkHRR from 19.4 to 15.1 kW/m^2^. With its higher GE content, F4 could not be ignited during testing and showed slow heat release without a clear pkHRR, indicating excellent flame-retardant properties. F0 film burned rapidly and thoroughly according to HRR curve data and observations (Figure 9), whereas F2 and F4 films showed continuous heat release under flameless conditions while maintaining their shapes even after testing. The GE aggregate group was nonflammable but underwent slow oxidation pyrolysis during heating, resulting in higher THR values for the composite films. Moreover, the extremely low pkSPR and TSP values observed here correspond to lower smoke risks and longer escape times in fire situations [39], since little burned ash was released into the air.

When these composite films underwent combustion, cellulose close to their surfaces burned first; then, internal cellulose thermally oxidized and decomposed under a slower process. Generated gas from internal pyrolysis resulted inthe appearance of some bumps on F2 film surfaces, but the smooth surface of F4 film was maintained due to internal pyrolysis suppression (Figure 9). GE nanoplates possessed highly specific surface areas and excellent thermal conductivities; with higher GE/cellulose ratios, denser laminated “labyrinths” formed in the films. This not only prevented oxygen from entering into film interiors but also conducted and dispersed a lot of heat from the films, thus reducing pkHRR of the composite films, preventing their combustion, and restraining internal pyrolysis. In addition, GE-constructed “labyrinths” adsorbed flammable organic volatiles from internal pyrolysis and prevented their release during combustion, thus reducing smoke generation. Therefore, pkSPR and TSP values of these composite films were low.

The effect of GE nanoplates on flame retardancy of the films was also confirmed by LOI values displayed in Table 3. The LOI value of pure cellulose film was only 17%, indicating high flammability. With increased GE content, the LOI of films increased to 29% for F2 and 38% for F4, which demonstrated that composite films exhibit improved flame retardancy.

The cellulose/GE composite films tested possessed excellent EMI shielding, good conductive properties, thermal stability, and flame resistance. They should be further investigated and considered as potential candidates for multipurpose materials in various applications, such as electronics and radar evasion.

## 4. Conclusions

Cellulose/GE composite films, with improved EM shielding and flame retardancy, were fabricated by introducing GE nanoplates into a cellulose matrix. GE nanoplates were added before cellulose dissolution in an ionic liquid ([Amim]Cl), and composite films were obtained by regeneration in a coagulation bath and controlled drying. GE introduction formed stacked laminated structures in the dried films due to the controlled shrinkage of the cellulose matrix, with laminated regularity dependent on the degree of film shrinkage. EMI SE and electrical conductivity of these composite films increased with increased GE content, while fluctuations in SE with frequency reduced. SE at 1500 MHz for the F4 film reached 22.3 dB, which is considered an adequate and moderate level of EM shielding for many applications. TG analysis indicated that these composite films exhibited improved thermal stability due to GE addition. Reduced flammability was observed from the extended TTI, and reduced pkHRR values were derived from cone calorimetry tests in addition to increased LOI values. In particular, the F4 film could not be ignited by direct ignition or during cone calorimetry testing, thus exhibiting outstanding flame resistance. The improved EM shielding and improved flame retardancy observed in these composite films derive from their stacked laminated structures of embedded GE nanoplates, making them suitable for consideration as potential candidates for multipurpose materials in electronics, radar evasion, and other applications.

## Figures and Tables

**Table 1:** General properties of cellulose and composite films.

Film Samples	F0	F1	F2	F3	F4
Wet thickness (mm)	1.78	1.81	1.81	1.88	1.93
Dry thickness (mm)	0.04	0.06	0.09	0.13	0.17
Planar density (g/m^2^)	46.2	64.9	104.1	133.7	161.2
Tensile strength (MPa)	69.4	54.1	39.7	25.8	11.3
Elongation at break (%)	5.4	4.3	3.7	2.7	2.9

**Table 2:** Comparation of typical cellulose-based films for EM shielding.

Materials	Method	Frequency (GHz)	EMI SE (dB)	Specific SE (dB·cm^2^/g)	Reference
Lotus fiber/GE (1/2) film	Regeneration	8.2–12.4	8.1	-	[33]
Cellulose/graphite (1/2) film	Regeneration	1.5	17.4	-	[27]
Cellulose/GE (1/2) film	Regeneration	1.5	22.3	1381.8	This work
Cellulose nanofiber/GE film	Vacuum filtration	8.2–12.4	27.4	5700	[31]
Cellulose aerogel/GE film	Hot-pressing	8.2–12.4	47.5	1384.2	[26]

**Table 3:** Cone calorimeter test results and LOI values of cellulose and composite films.

Film Sample	F0	F2	F4
TTI/TTO (s)	5/10	10/14	Unignited
pkHRR (kW/m^2^)	19.4	15.13	5.29
THR_0–600_ (MJ/m^2^)	0.63	2.85	2.03
pkSPR (m^2^/s)	<0.01	<0.01	<0.01
TSP_0__–600_ (m^2^)	<0.1	<0.1	<0.1
LOI (%)	17	29	38

## Data Availability

The data presented in this study are available in the article.
